# Lipid Droplet Dynamics are Modulated by Age and APOE Genotype

**DOI:** 10.1002/alz.091341

**Published:** 2025-01-03

**Authors:** Isaiah O Stephens, Diksha Satish, Cassi Friday, Katy Smith, Nicholas A Devanney, Ana Maria L/K Cornea, Scott Gordon, Lance A Johnson

**Affiliations:** ^1^ University of Kentucky, Lexington, KY USA; ^2^ Sanders‐Brown Center on Aging, Lexington, KY USA; ^3^ Department of Physiology, Lexington, KY USA; ^4^ Paul Laurence Dunbar High School, Lexington, KY USA; ^5^ Saha Cardivascular Research Center, Lexington, KY USA

## Abstract

**Background:**

Aging microglia accumulate lipid droplets (LDs), secrete pro‐inflammatory cytokines, and are defective in phagocytosis. The E4 allele of Apolipoprotein E (*APOE*) is the strongest genetic risk factor for late‐onset Alzheimer’s disease (LOAD) and is associated with increased neuroinflammation and LD accumulation. Here, we aimed to determine if the effects of aging and the E4 allele are synergistic in causing the accumulation of LDs seen in LOAD. Specifically, we hypothesize that *APOE* genotype modulates the LD proteome and lipidome at baseline and with inflammation. Additionally, we show the effects of *APOE* genotype and age on LD accumulation in monocyte‐derived human macrophages treated with sex‐matched exogenous serum.

**Method:**

Primary microglia were isolated from mice expressing human ApoE3 and ApoE4. Microglia were exposed to oleic acid (OA), LPS, OA+LPS, dead N2A cells, or dead N2As+LPS to stimulate an inflammatory response, then lipid droplet content was analyzed. To examine the impact of inflammation on LDs *in vivo*, ApoE3 and ApoE4 mice were injected with saline or LPS and liver tissue collected at 24h. Density gradient centrifugation was used for isolation of LD‐enriched fractions and these were analyzed using mass spectrometry proteomics. To determine the impact of age on circulating factors that may affect LD dynamics, human Peripheral Blood Mononuclear Cells (PBMCs) were treated with serum from different aged donors and the impact on LDs was analyzed.

**Result:**

Primary microglia from ApoE4 mice accumulated significantly more LDs at baseline, with OA, LPS, and N2As as a percentage of E3 control. Proteomics revealed that LD fractions from E4 mice are enriched for proteins involved in innate immunity, while E3 LDs are enriched for lipid b‐oxidation proteins. Lipidomics showed an increase in phosphatidylcholine distribution in the LD membrane of E4‐control and LPS‐treated droplets. Finally, a significant positive correlation between the age of serum samples used to treat human macrophages and LD accumulation in the PBMCs was observed.

**Conclusion:**

In agreement with prior studies, E4 microglia accumulate more LDs compared to E3 microglia under all conditions tested. The proteomic profile of E4 LDs support the hypothesis that E4 expression increases inflammation under basal and stimulated conditions, causing a more robust response.

